# Accuracy of formula-based volume and image segmentation-based volume in calculation of preoperative cystic jaw lesions’ volume

**DOI:** 10.1007/s11282-023-00731-5

**Published:** 2023-12-19

**Authors:** Yasmein Maher El-beblawy, Ahmed Mohamed Bakry, Maha Eshaq Amer Mohamed

**Affiliations:** Present Address: Department of Oral and Maxillofacial Radiology, Faculty of Dentistry, Minia University, Shalaby Street, Minya, Egypt

**Keywords:** Jaw cyst, Volume analysis, Ellipsoid formula

## Abstract

**Objective:**

The aim of this study was to assess the accuracy of formula-based volume measurements and the 3D volume analysis with different software packages in the calculation of preoperative cystic jaw lesions’ volume. The secondary aim was to assess the reliability and the accuracy of 3 imaging software programs for measuring the cystic jaw lesions' volume in CBCT images.

**Materials and methods:**

This study consisted of two parts: an in vitro part using 2 dry human mandibles that were used to create simulated osteolytic lesions to assess the accuracy of the volumetric analysis and formula-based volume. As a gold standard, the volume of each bone defect was determined by taking an impression using rapid soft silicone (Vinylight) and then quantifying the volume of the replica. Afterward, each tooth socket was scanned using a high-resolution CBCT. A retrospective part using archived CBCT radiographs that were taken from the database of the outpatient clinic of the oral and maxillofacial radiology department, Faculty of Dentistry, Minia University to assess the reliability of the 3 software packages. The volumetric data set was exported for volume quantification using the 3 software packages (MIMICS-OnDemand and InVesalius software). Also, the three greatest orthogonal diameters of the lesions were calculated, and the volume was assessed using the ellipsoid formula. Dunn’s test was used for pair-wise comparisons when Friedman’s test was significant. The inter-examiner agreement was assessed using Cronbach’s alpha reliability coefficient and intra-class correlation coefficient.

**Results:**

Regarding the results of the retrospective part, there was a statistically significant difference between volumetric measurements by equation and different software (*P* value < 0.001, Effect size = 0.513). The inter-observer reliability of the measurements of the cystic lesions using the different software packages was very good. The highest inter-examiner agreement for volume measurement was found with InVesalius (Cronbach’s alpha = 0.992). On the other hand, there was a statistically significant difference between dry mandible volumetric measurements and Gold Standard. All software showed statistically significantly lower dry mandible volumetric measurements than the gold standard.

**Conclusion:**

Computer-aided assessment of cystic lesion volume using InVesalius, OnDemand, and MIMICS is a readily available, easy to use, non-invasive option. It confers an advantage over formula-based volume as it gives the exact morphology of the lesion so that potential problems can be detected before surgery. Volume analysis with InVesalius software was accurate in determining the volume of simulated periapical defects in a human cadaver mandible as compared to true volume. InVesalius software proved that open-source software can be robust yet user-friendly with the advantage of minimal cost to use.

## Introduction

Odontogenic cysts are the most frequent lesions appearing in the jaws. They are defined as cavities filled with liquid, semiliquid, or gaseous content with odontogenic epithelial lining and connective tissue on the outside. They originate from the epithelial component of the odontogenic apparatus or its remnants that lie entrapped within the bone or in the peripheral gingival tissues [1].

Most cysts of the jaws are discovered incidentally on panoramic radiographs or they destroy surrounding structures and cause problems such as loosening of teeth or facial deformity. Panoramic radiograph is often routinely used as a primary diagnostic tool for the detection of cystic lesions, particularly in follow-up to assess neo-ossification. In more complex maxillofacial surgical cases requiring 3D information of the region of interest, CBCT offers advantages over conventional 2D imaging modalities, such as a detailed representation of cysts in bone tissue and the involvement of surrounding structures, such as tooth roots and nerves. For this reason, they allow surgeons to accurately plan surgical management [2].

According to cyst size, jaw cysts can be classified into small, median, and large mandibular cysts, which often invade teeth and can seriously affect the quality of life. Cysts tend to enlarge and grow, leading to resorption of bone tissue. Depending on the degree of resorption, cysts may cause severe damage such as bone fractures. Treatment planning for cysts depends on cyst location, size, extent of tissue damage, availability of surgical access, patient’s age, proximity of the cyst to vital structures, and significance of the affected teeth in terms of eruption. Marsupialization or decompression is the first consideration if the lesion invades adjacent structures or if primary enucleation could cause pathological fractures or neurological damage [3, 4].

Small cysts can generally achieve satisfactory results after root canal treatment, while curettage is an effective and radical treatment for median and large cysts. However, large cysts (> 4 cm in diameter) lead to a large area of involvement and more critical anatomical damage and are more likely to cause problems, such as bone destruction, maxillofacial deformity, and can affect occlusal function. Therefore, conventional cyst curettage is not effective enough [5].

Non-invasive determination of the volume of the lower jaw cysts is a helpful additional process in the preoperative diagnosis. In this way, a geometrical approximated volume can be calculated. Linear measurements on CBCT images are possible in all three planes and directions and are employed in routine practice. Volumetric studies on bone regeneration of cystic cavities were carried out using CT scanning and measuring the three maximum diameters of the cavity. Nonetheless, it is still an approximated volume, not a real volumetric measurement. From these diameters, approximate volumes were calculated using the cubic and ellipsoid formulas. The data suggest that maximum tumor diameter-based size characterization, especially the cuboid formula and the maximum diameter alone, should not be recommended [6].

Image segmentation is used to analyze and process 2D or 3D images to achieve extraction, 3D reconstruction, and 3D visualization of anatomical structures or anomalies such as tumors or cysts. Volumetric analysis requires segmentation of an object, such as a tooth, from its surrounding structures. With the help of image segmentation, the physician is provided with a tool to determine the volume of a jaw lesion, and, in addition to that, the anatomical extent can be clearly defined and used for surgical planning. The volume of affectation of caries is determined with the k-mean grouping method and the threshold method, the latter being the most recommended [7].

Volumetric analysis within the field of dental–maxillofacial radiology can be utilized for assessing volumetric estimates of different bone injuries counting; periapical abscesses, cysts, and tumors. Identifying the volume of a lesion is vital, particularly in comparing the measurements with the follow-up radiographs. The error of volumetric measurement in CBCT reconstruction may have an important clinical impact. The inaccuracy of volumetric measurement can influence superimposition and comparison before and after surgery [8, 9].

A growing number of software programs to manage and analyze Digital Imaging Communications in Medicine (DICOM) files are available in the market every year. Many of these have incorporated tools for segmentation and volumetric analysis. Several software packages already provide clinicians with a dedicated tool for assessing the volumes of regions of interest in cubic millimeters. Several previous studies have focused on the accuracy of volume measurements of teeth from CBCT data [10, 11]. Therefore, is the formula-based volume of cystic lesions comparable to values to the volumetric analysis values? Is the volumetric analysis of cystic jaw lesions affected by the change in software?

Thus, the aim of this study was to assess the accuracy of formula-based volume measurements and 3D volume analysis with different software packages in the calculation of preoperative cystic jaw lesions’ volume. The secondary aim was to assess the reliability and the accuracy of 3 imaging software programs for measuring the cystic jaw lesions' volume in CBCT images.

## Materials and methods

Approval for this study was granted by the Research Ethics Committee (REC) of the Faculty of Dentistry, Minia University, under approval number 534—1/11/2021.

1. In vitro part of the study
  This part constituted two dry human mandibles which were provided by the Department of Anatomy, Faculty of Medicine, Minia University. In the cancellous bone at the base of the extraction sockets, bone defects were cut and equally distributed in different tooth positions, with dental burs to mimic bone lesions. The bone defects were of different dimensions and shapes. To simulate the attenuation caused by soft tissue in an in vivo situation, a wax sheet (Base Plate Wax Cavex, Modelling Wax. Netherlands) with dimensions of (175 × 80 × 1.5) mm was used. The sheets were placed sequentially one behind another in increasing numbers, gently pressed together to avoid any empty space between them but without deforming the wax.
  (I) Physical volume measurements (gold standard):
    For the experiment, as a gold standard, the volume of each created bone defect was determined by taking an impression and creating a replica Rapid soft silicone (Vinylight, BMS DENTAL, Italy) impression material and then quantifying the volume. The internal surface of each bone defect was first coated with a thin layer of melted prosthetic dental wax facilitating the separation of the replica from the bone defect. Impressions of these holes were placed with a carrier directly into the defects through the extraction sockets.
    The impressions of the artificial bone defects were weighed using an electronic analytical balance (Shimadzu Corporation Aty224, Kyoto, Japan) with an accuracy of 0.0001 g (Fig. 1). As this electronic balance had a fully automated calibration technology and a micro-weighing scale, the values of all samples were accurately measured. Each mounted sample was cleaned and dried with tissue paper before weighing. To ensure accuracy, the balance was kept on a free-standing table at all times—away from vibrations—and weighed the specimens with the glass doors of the balance closed to avoid the effect of air. Each volume was then obtained by multiplying the weight by the density of the impression material [4, 8]. The data were collected and reported in a chart.

**Fig. 1:**
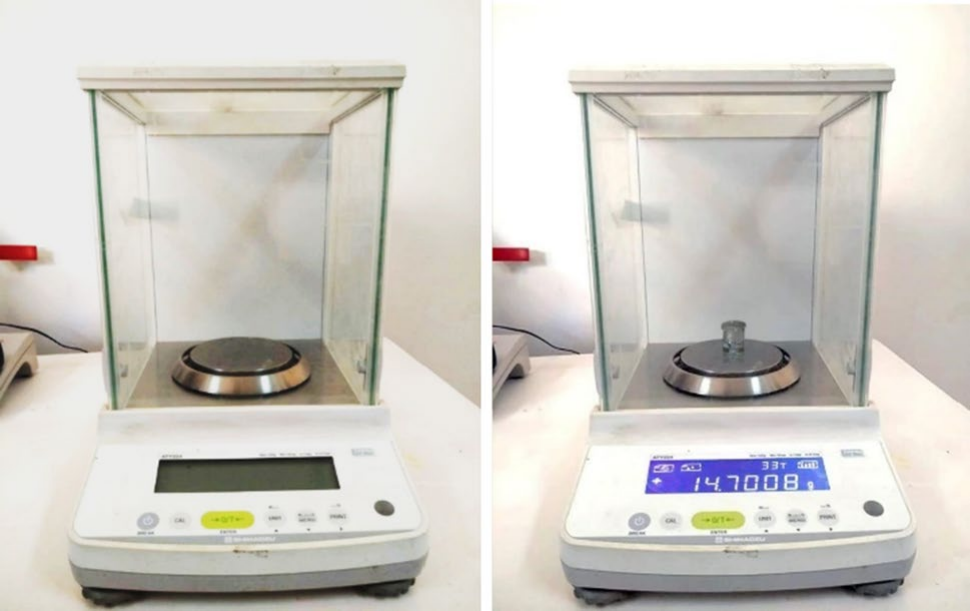
Analytical balance
  (II) CBCT examination:
    Screening radiographs and CBCT scans were taken to confirm that there were no existing periapical lesions associated with the assessed teeth. The examinations were carried out using a standardized method to ensure the repeatability of the examination and the reliability of the analysis of the artificial lesions was used. CBCT examinations of the simulated pathosis were performed using **(**SCANORA^®^ 3Dx, Soredex, Helsinki, Finland) with 10 mA and 90 kV, FOV 50 × 100 mm, 3 s of effective exposure time, focal spot 0.5 mm, and isotropic voxel size of 0.4 × 0.2 mm. Mimics, OnDemand, and InVesalius software were used for the volume detection of bone defects.
  (III) The formula-based volume of bone defects:
    Maximum bone defect diameters were assessed using OnDemand software in millimeters (mm), mesiodistal, buccolingual, and coronal–apical directions in axial and cross-sectional CBCT cuts. The approximate cyst volume was calculated using the ellipsoid formula [5, 12–14]:
    $${\text{VOL}}=\pi \times \frac{(x\times y\times z)}{6},$$
    *X*: Coronal plane (maximal width/bucco-lingual direction). *Y*: Axial plane (maximal length/mesio-distal direction). *Z*: Sagittal plane (maximal height/coronal–apical direction).
2. Retrospective part of the study
  The second part was retrospective and included 49 radiographic images of cystic jaw lesions from the archived database of the Oral and Maxillofacial Radiology Department Faculty of Dentistry, Minia University, in the period from January 1, 2020 to December 31, 2022 and met the inclusion criteria. Radiographic images with well-defined osteolytic lesions were included. Radiographic images were excluded from the study based on several criteria; patients with neoplastic jaw lesions, lesions with ill-defined borders, lesions with large extra-osseous extensions to the soft tissue, patients who have metallic implant bodies or metallic crowns, and images with low-quality radiographs. Clinicopathological data were collected from medical records as well as pathology and surgery reports.
  All these images were taken using the same CBCT imaging system (SCANORA^®^ 3Dx, Soredex, Helsinki, Finland) with 4–10 mA and 60–90 kV, 18–34 s of exposure time, focal spot 0.5 mm. The acquired data from the X-ray machine were exported using a specific type of file [Digital Imaging and Communication in Medicine (DICOM)]. Thereby generated data can be extracted and further processed with the help of biomedical image processing software, for image segmentation purposes.
  (A) Data collection
    Type of cystic lesion, lesions’ location (maxilla vs. mandible), and region (anterior vs. posterior) were detected.
  (B) Formula-based volume of cystic lesions:
    Maximum cyst diameters were assessed, in millimeters (mm), in mesiodistal, buccolingual, and coronal–apical directions (Fig. 2) in axial and cross-sectional CBCT cuts using OnDemand software.
    The approximate cyst volume was calculated using the ellipsoid formula.

**Fig. 2:**
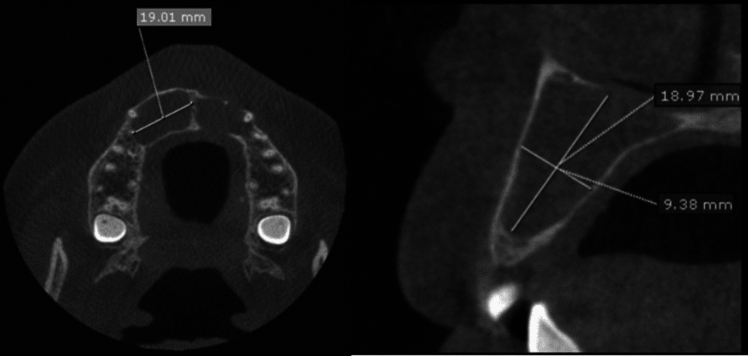
Maximum lesion dimensions in MD/BL/CA directions
  (C) Image segmentation and volume analysis
    This study compares OnDemand, Mimics, and InVesalius which were compatible with the Windows operating system. Segmentations were performed according to each software manufacturer’s recommendations and using the interactive threshold technique, meaning that the operator selected the best threshold interval for visualizing the entirety of the anatomic boundaries of the cystic lesion (Fig. 3). To investigate the reliability of the employed volume calculation procedure, five randomly selected patients were measured twice by three investigators to analyze inter-observer reliability. All readers were blinded to each other’s results.

**Fig. 3:**
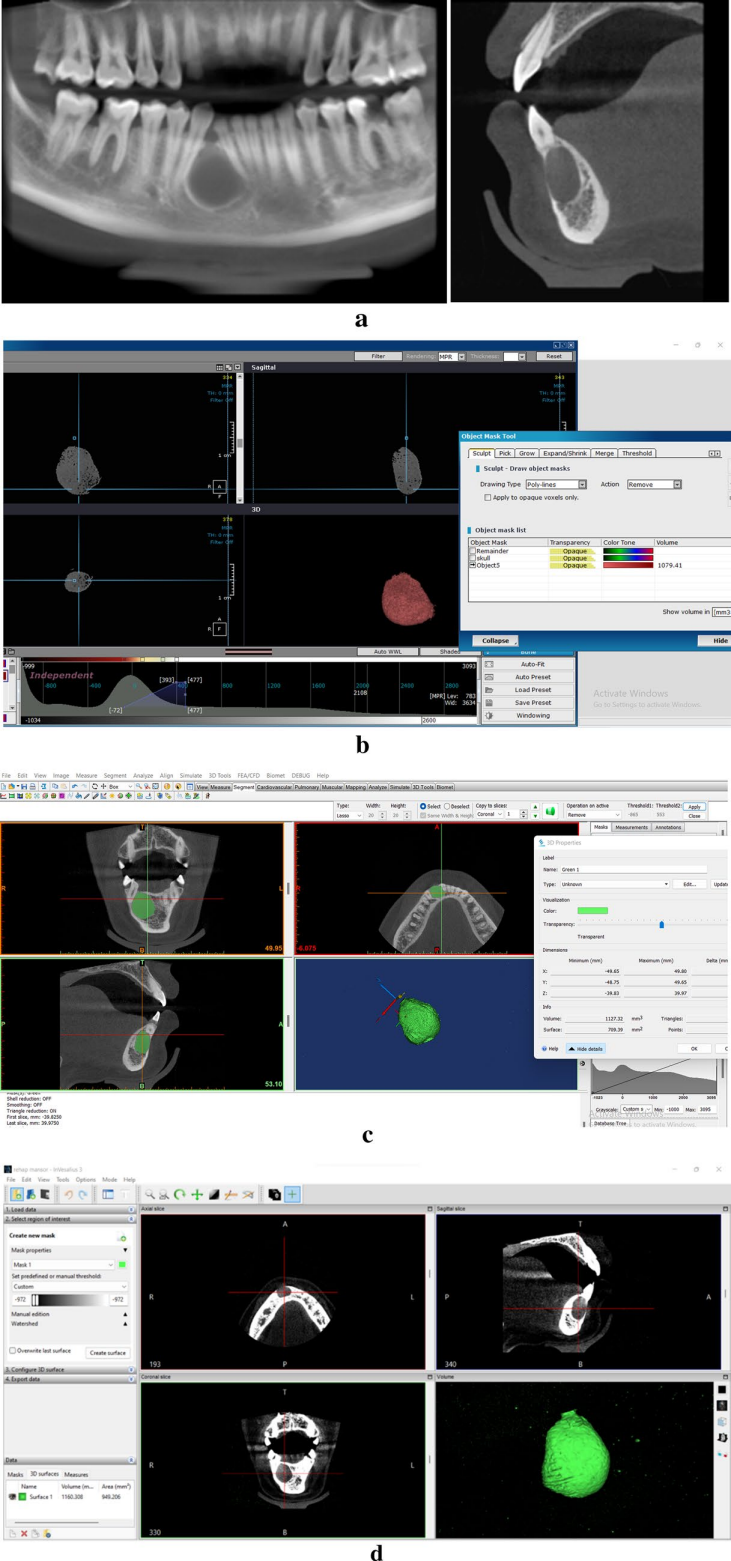
CBCT reformatted panoramic view (**1a**) and sagittal cut of a lateral periodontal cyst (**2a**), OnDemand volume analysis of the cystic lesion (**b**), MIMICS volume analysis of the cystic lesion (**c**), InVesalius volume analysis of the cystic lesion (**d**)

### Statistical analysis

Numerical data were explored for normality by checking the distribution of data and using tests of normality (Kolmogorov–Smirnov and Shapiro–Wilk tests). All data showed non-normal (non-parametric) distribution. Data were presented as median, range, mean, and standard deviation (SD) values. Friedman’s test was used to compare between gold standard and different software. Dunn’s test was used for pair-wise comparisons when Friedman’s test was significant. The inter-examiner agreement was assessed using Cronbach’s alpha reliability coefficient and intra-class correlation coefficient (ICC). Closer values of these coefficients to one indicate better inter-observer agreement. Qualitative data were presented as frequencies and percentages. The significance level was set at *P* ≤ 0.05. Statistical analysis was performed with IBM SPSS Statistics for Windows, Version 23.0. Armonk, NY: IBM Corp.

## Results

1. In vitro part
  - Comparison between gold standard and different software
    There was a statistically significant difference between dry mandible volumetric measurements and gold standard (*P* value < 0.001, Effect size = 0.828). Pair-wise comparisons revealed that there was no statistically significant difference between Mimics, OnDemand, and InVesalius modalities. All showed statistically and significantly lower dry mandible volumetric measurements than gold standard. Volumetric measurement by equation showed the statistically significantly lowest value.
  - Comparison between absolute errors of different software
    Absolute error was calculated as Measurement by modality—Gold Standard.
    There was a statistically significant difference between absolute errors of dry mandible volumetric measurements of different modalities (*P* value < 0.001, Effect size = 0.769). Pair-wise comparisons revealed that the equation showed the statistically significantly greatest absolute error in dry mandible volumetric measurements. There was no statistically significant difference between Mimics and OnDemand. Both showed statistically significantly lower absolute error. InVesalius showed the statistically significantly lowest absolute error indicating that it is the most accurate modality.
  - Comparison between relative errors of different software
    Relative error was calculated as Absolute error/Gold Standard × 100.
    There was a statistically significant difference between relative errors of dry mandible volumetric measurements of different modalities (*P* value < 0.001, Effect size = 0.769). Pair-wise comparisons revealed that the equation showed the statistically significantly greatest relative error in dry mandible volumetric measurements. There was no statistically significant difference between Mimics and OnDemand. Both showed statistically significantly lower relative error. InVesalius showed the statistically significantly lowest relative error indicating that it is the most accurate modality (Table 1 and Fig. 4).

**Table 1:** Descriptive statistics and results of Friedman’s test for comparison between relative errors of dry mandible volumetric measurements (%) by different software

Modality	Median	Minimum	Maximum	Mean	SD	*P *value	Effect size (w)
Mimics	− 8.2^B^	− 9.8	− 2.3	− 6.7	3.2	< 0.001*	0.769
OnDemand	− 9^B^	− 10	3.4	− 7	4.5		
InVesalius	− 4.3^C^	− 9.4	− 1.9	− 4.7	2.4		
Equation	− 22.7^A^	− 43.8	− 15.2	− 25.8	9.1		

*Significant at *P* ≤ 0.05, different superscripts indicate statistically significant difference

**Fig. 4:**
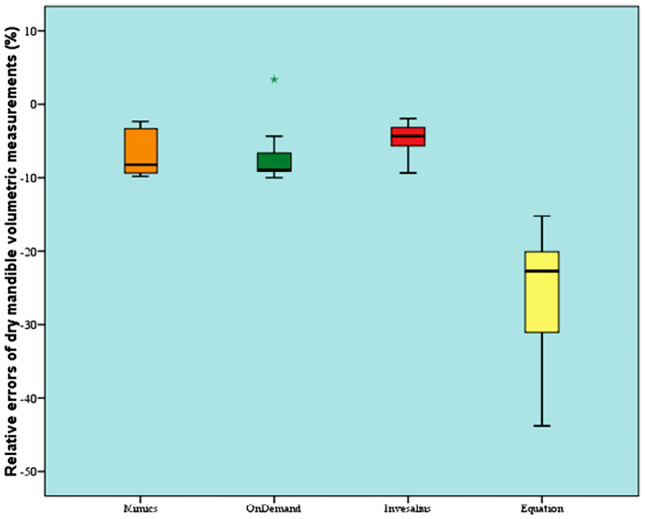
Box plot representing median and range values for relative errors of dry mandible volumetric measurements by different software (star represents outlier)
2. Retrospective part
  1. Demographic data
    The present study was conducted on 49 patients: 26 males (53.1%) and 23 females (46.9%). The most common type of cyst was radicular cyst (36.7%) followed by odontogenic keratocyst (20.4%). Almost half of the cysts (51%) were found in the lower arch and 49% were in the upper arch.
  2. Volumetric measurements (mm^3^)
    - Comparison between measurement by equation and different software
      There was a statistically significant difference between volumetric measurements by equation and different software (*P* value < 0.001, Effect size = 0.513). Pair-wise comparisons revealed that there was no statistically significant difference between Mimics, OnDemand, and InVesalius software. All showed statistically significantly higher volumetric measurements than measurements by equation (Table 2 and Fig. 5).

**Table 2:** Descriptive statistics and results of Friedman’s test for comparison between linear volumetric measurements (mm^3^) by equation and different software

Modality	Median	Minimum	Maximum	Mean	SD	*P* value	Effect size (w)
Equation	1161.9^B^	80.1	12,813.1	2020.5	2653.9	< 0.001*	0.513
Mimics	1430.8^A^	96.9	22,471	3244.3	4805.7		
OnDemand	1567.6^A^	88.9	22,192.9	3209.2	4714.3		
InVesalius	1512.4^A^	116.9	21,333.7	3225.9	4751.9		

*Significant at *P* ≤ 0.05, Different superscripts indicate statistically significant difference

**Fig. 5:**
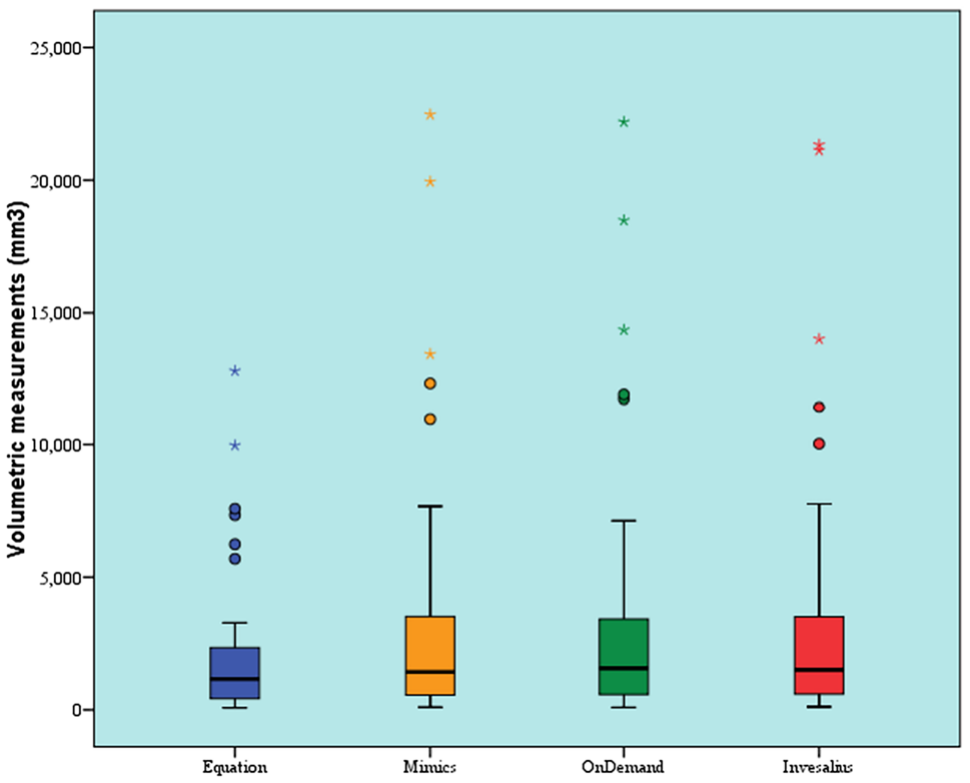
Box plot representing median and range values for volumetric measurements by equation and different software (Stars and circles represent outliers)
    - Inter-examiner agreement
      All software showed very good inter-examiner agreement. The highest inter-examiner agreement for volume measurement was found with InVesalius (Cronbach’s alpha = 0.992). This was followed by Mimics (Cronbach’s alpha = 0.989). The lowest agreement was found with OnDemand (Cronbach’s alpha = 0.963) (Table 3).

**Table 3:** Results of Cronbach’s alpha reliability coefficient and Intra-Class Correlation Coefficient (ICC) to assess inter-examiner agreement

Modality	Cronbach’s alpha	ICC	95% Confidence interval for ICC
Mimics	0.989	0.979	0.413–0.999
OnDemand	0.963	0.928	0.185–0.998
InVesalius	0.992	0.984	0.518–1

## Discussion

Researchers have utilized linear measurements of the CBCT scans for determining the volumes of periapical defects. Linear measurements provide limited information and values in one plane in comparison to volumetric measurements. As the periapical lesions may have irregular or 3D shapes, the algorithm for calculating the volume of the sphere cannot be applied. Linear accuracy of the measurements done by this method is inadequate to translate into a clinical setting [15–17].

In this study, the DICOM data from CBCT scans were segmented using OnDemand, MIMICS, and InVesalius software. OnDemand is the software on a local workstation. MIMICS was chosen because of its widespread use in Biomedical engineering. MIMICS software provides semi-automatic segmentation and interpolation between slices and detects the margins to save time. InVesalius software was included according to its easy availability for everybody on a free open-source basis.

In this study, there was a statistically significant difference between volumetric measurements by equation and different modalities (*P *value < 0.001). All software showed statistically significantly higher volumetric measurements than measurements by equation. This is consistent with Kauke et al. [5] who investigated the agreement and overlap between image segmentation and formula-based volume approximation. The ellipsoid formula yielded volume approximations that were in mean 10.1% lower when compared to segmentation-based volume approximations using ITK-snap software. They inferred that formula-based volume approximation is error-prone and not precise when compared to image segmentation as odontogenic jaw lesions rarely grow in the perfect shape of a cuboid or ellipsoid, both formulas are naturally error-prone. In particular, this applies to infiltrative odontogenic neoplasms, capable of arbitrary three-dimensional infiltration with budding and thus irregular three-dimensional configuration.

This can be explained by Lizio et al. [18] who stated that calculating the area of a lesion as a regular ellipsoid is an approximation that does not take into consideration the frequent morphological irregularities of these lesions, especially keratocyst, and the presence of collateral cavities and scalloped contours. Its shortcomings include inaccurate discrimination of cyst border and the inability to assess the cyst’s relation with the surrounding vital structures. Furthermore, the ellipsoid formula depends on bi-dimensional evaluation and measurements of the largest dimensions of the diameter and depth of the lesion. Observer performance, selection of reference points, mouse sensitivity, and software capabilities are all important factors in the measurement of lesion dimensions. In this study, experienced and calibrated oral radiologists equally familiar with the software used acted as operators [18, 19].

On the other hand, Dejaco et al. [12] found that an ellipsoid formula using the largest diameter of a lesion in all three planes provided a reasonable approximation of head and neck tumor volumes when compared to manual slice-by-slice segmentation. However, the use of such a mathematical formula can be cumbersome in multi-locular lesions. Also, Sacher et al. [20] who used OsiriX software, concluded that using the formula is easy to use and allows for an accurate and precise prediction of the amount of time needed for bone regeneration after both cystostomy and cystectomy. This means that the formula can be used for comparable conditions.

Concerning the operator-dependent error, manual and semi-automatic segmentation showed very good inter-operator reliability according to the ICC values and the small volumetric differences found between the three recordings. The highest inter-examiner agreement for volume measurement was found with manual segmentation using InVesalius software (Cronbach’s alpha = 0.992). This was followed by semi-automated segmentation using MIMICS software (Cronbach’s alpha = 0.989) and OnDemand software (Cronbach’s alpha = 0.963).

In this respect, the segmentation process delegated most of this task to the software algorithm, which reduced the magnitude of the observer-related error. For the same reason, semi-automatic segmentation almost voided the difference between the readings performed by three observers with different level of expertise in 3D imaging. In addition, threshold adjustment is solely dependent on the operator; thus, checking the integrity of the segmented object on 3 spatial planes is crucial. Although the aforementioned threshold adjustments could have created differences between the operators, the results of this study still represent high inter-operator. It was concluded that the selection of threshold sensitivity values was not reliable [21].

Also, Weissheimer et al. [10] compared the precision and the accuracy of 6 imaging software programs (Mimics, Dolphin3D, Ondemand3D and ITK-Snap, and InVivo Dental). The method repeatability for the patients' oropharynx measurements was high (ICC 0.0.94) for 6 imaging software programs. In addition, Chen et al. [22] assessed the reliability and accuracy of three different commercially available software packages (Amira, 3Diagnosys, and OnDemand3D). The intra- and inter-observer reliability of the measurements using all three software packages were excellent (ICC ≥ 0.75). All three software packages generally underestimated the upper airway volume. In Abdelhamid et al. [23, 24] study, the inter-observer reliability was high for OnDemand and InVesalius programs, which indicated minimal subjective variance for well-trained practitioners. Also, ElShebiny et al. [25] reported high reliability was observed between four tested software packages including OnDemand3D for intra-operator and inter-operator values.

In this study, MIMICS software measurements showed higher mean volumes (3244.3 mm^3^) than OnDemand (3209.2 mm^3^). This is consistent with Weissheimer et al. [10] also assessed segmentation with interactive thresholding using 6 software including MIMICS and OnDemand. The volume measurements with the 6 imaging software were statistically different (*P* = 0.006). The descriptive statistical analysis showed higher oropharynx mean volumes for MIMICS and lower mean volumes for Ondemand3D. There were no statistically significant differences (*P* > 0.05) among ITK-Snap, Mimics, OsiriX, Dolphin3D, and Ondemand3D.

These results are in good agreement with a previous study by El H & Palomo. [26] who reported that OnDemand3D software sometimes fails to depict certain parts of the upper airway, which subsequently leads to an underestimation of the airway volume. This phenomenon could originate from the CBCT image acquisition process and/or the subsequent image segmentation by means of thresholding. During CBCT image acquisition, anatomical structures are discriminated based on their radiographic density. However, voxels residing on tissue boundaries can contain more than one tissue type. This phenomenon is known as the partial volume effect. The result of the partial volume effect is that voxels are erroneously allocated to “soft tissue” instead of “air” and hence “upper airway” during the image segmentation process [24, 27].

This may be explicated by Lo Giudice et al. [21] who inferred that software based on a threshold-based segmentation algorithm (MIMICS, OnDemand, and InVesalius) could cause an under/overestimation of boundaries since the segmentation procedure still relies on the operator visual discrimination of the bony structure and definition of threshold-level. Consequently, if an accurate definition of an object’s boundaries is required, highly skilled clinicians can perform manual refinement.

In this study, pair-wise comparisons revealed that there was no statistically significant difference between Mimics, OnDemand, and InVesalius software. Abdelhamid et al. [23] also showed that OnDemand and InVesalius software had comparable volumetric computation in the presurgical volumetric analysis in secondary alveolar cleft bone grafting, but InVesalius was relatively faster than On-Demand 3D. To select the best option, it is necessary to analyze not only the time spent in the process, knowing that time is important, but accuracy and robustness of the software [28]. In addition, Ghoneim and Gad [29] tested the difference between measures carried out by MIMICS and AutoCAD software for the post-marsupialization of cystic lesions, and the results were non-significant (*P* > 0.05).

In conclusion, volumetric measurements are influenced by the software's imaging processing methods and segmentation techniques, and these differ between the different software. This current study shows that the 3D computer-aided assessment of cyst volumes provides information that is accurate enough to be used for preoperative planning. In addition, further investigations of volumetric analysis of cystic jaw lesions will be needed to correlate with different shapes of cystic lesions. Also, further investigation of the effect of the preoperative size of cystic jaw lesions and proper grouping of the sizes will be valuable.

## Data Availability

Data available on request due to privacy/ethical restrictions.
